# Not Only Caseload but Also Patient Selection Is Predictive of Mortality After Pancreatic Resection

**DOI:** 10.1097/AS9.0000000000000536

**Published:** 2024-12-30

**Authors:** Rene Mantke, Barbara Seliger, Shuji Ogino, Markus W Büchler, Richard Hunger

**Affiliations:** From the * Department of General Surgery, University Hospital Brandenburg, Brandenburg Medical School Theodor Fontane, Brandenburg, Germany; † Faculty of Health Sciences Brandenburg, Brandenburg Medical School Theodor Fontane, Brandenburg, Germany; ‡ Institute for Translational Immunology, Brandenburg Medical School Theodor Fontane, Brandenburg, Germany; § Department of Epidemiology, Harvard T.H. Chan School of Public Health, Harvard University, Boston, MA; ‖ Program in MPE Molecular Pathological Epidemiology, Department of Pathology, Brigham and Women’s Hospital, Harvard Medical School, Boston, MA; ¶ Broad Institute of MIT and Harvard, Cambridge, MA; # Tokyo Medical and Dental University (Institute of Sience Tokyo), Tokyo Japan; ** Botton-Champalimaud Pancreatic Cancer Centre, Lisbon, Portugal; †† University of Heidelberg, Heidelberg, Baden-Württemberg, Germany.

**Keywords:** pancreatic centers, pancreatic surgery, quality assurance, volume-outcome-relationship

## Abstract

**Background:**

Centralization of pancreatic surgery in high-volume centers is regarded as a key strategy in improving the outcome quality. However, the specific factors, in addition to higher case volumes, that influence inhospital mortality remain unclear.

**Methods:**

In this retrospective observational study, the German nationwide diagnostic-related groups statistics were analyzed for 86,073 patients with pancreatic resections. Hospitals performing at least 50 resections per year were identified as high-volume pancreatic centers (HVPCs). Statistical analyses compared crude and adjusted estimates of inhospital mortality for patients treated in HVPCs and non-HVPCs. A generalized mixed model was used for risk adjustment, considering various factors such as age group, sex, diagnosis, and comorbidities (ClinicalTrail.gov, NCT06390891).

**Results:**

A total of 24.2% (n = 20,798) of all pancreatic resections were performed in 23 HVPCs. The crude inhospital mortality for all patients undergoing resection was 9.0%. Crude inhospital mortality in HVPCs was 5.5% compared with 10.1% in non-HVPCs (*P* < 0.001). HVPCs performed more complex resections including more concomitant procedures. On the other hand, HVPCs treated younger patients and patients with less complicated comorbidities. Statistical adjustment of comorbidities and patient characteristics resulted in a significant increase of inhospital mortality from 5.5% to 8.7% in HVPCs.

**Conclusions:**

HVPCs have significantly lower inhospital mortality than the other hospitals. Nevertheless, the superior quality of outcome can be attributed not only to the enhanced expertise of the centers but also, at least in part, to a healthier patient population on average. However, the extent to which this patient selection is due to active selection by the practitioners or other causes remains unclear.

## INTRODUCTION

Pancreatic surgery is a complex procedure that involves high rates of postoperative complications and significant hospital mortality.^1–5^ Furthermore, despite the progress made in the treatment of other forms of cancer, pancreatic cancer, which is a primary medical indication for pancreatic resection, has not experienced a notable improvement in survival rates over the past few decades.^6^ Centralization of pancreatic surgery in specialized centers is considered key to improving the quality of short- and long-term outcomes.^4,6^ This is supported by several studies, that have demonstrated an association between higher surgical volumes and lower hospital mortality rates, as well as improved survival.^1,7–10^ However, it is important to note that inhospital mortality may relate to multiple factors beyond the annual hospital caseload of pancreatic resections. These factors include, for instance, the quality of radiological diagnosis, appropriate indication for surgery, patient selection, selection of the appropriate surgical technique, the surgeon’s qualifications, the technical and structural conditions of the hospital, the presence of postoperative ERAS or Fast Track concepts, adequate identification and management of surgical complications, and the quality of the interdisciplinary team managing complications.^11,12^

Statistical multivariable adjustment techniques, such as logistic regression, are used for the assessment of volume-outcome relation to describe variances between hospitals.^9^ Although these techniques have limitations, they yield more reliable estimates than unadjusted values.^13,14^

Two observations, nonetheless, are important in contextualizing the results and findings of these studies. First, a recent review of 87 studies found an inverse relationship between the number of covariates used for risk adjustment and the benefit/advantage of higher procedure volume, suggesting that the volume effect may be overestimated.^11^ Second, upon informal review of a convenience sample of highly cited original articles (>300 citations) on the relationship between volume and outcome, a tendency was observed whereby particularly high-volume providers treated younger and less severely ill patients.^5,15,16^ These notions are consistent with findings that certain patient characteristics affect the likelihood of being treated in a high-volume hospital.^17–19^ To investigate this conundrum, the present study analyzed all hospitals in Germany with a high caseload of pancreatic resections (high-volume pancreatic centers [HVPCs]). For HVPCs, crude and adjusted inhospital mortality rates were contrasted and patient characteristics were compared with non-HVPCs.

We hypothesize that (A) more complex pancreatic resections were performed in HVPCs than in non-HVPCs and (B) HPVCs selected their patients more strongly than non-HPVCs due to their experience. In addition to the number of cases, this suspected selection could contribute to the low mortality rates of HVPCs. This question has not yet been investigated to date.

## METHODS

### Data

The German nationwide administrative diagnosis-related group statistic, provided by the Research Data Centers of the Federal Statistical Office and the Statistical Offices of the Federal States (Diagnostic Related Groups statistics 2010–2018), was queried by controlled remote data analysis. The dataset comprises all inpatient episodes of all German hospitals, except for military and psychiatric hospitals. All German hospitals must electronically report a specified set of patient variables to the Institute for the Remuneration System in Hospitals (“Institut für das Entgeltsystem im Krankenhaus”) due to legal obligation. The diagnoses are recorded according to a modified International Classification of Diseases System (version 10, German Modification) and procedures according to a modified version of the International Classification of Procedures in Medicine (Operationen- und Prozedurenschlüssel).

### Patient Selection

Patient demographics (sex and age), treatment details (main and additional diagnoses, medical procedures, admission, and discharge status), and a unique pseudonymized hospital identification number were extracted from the dataset. All patients who underwent partial or total pancreatic resections from 2010 to 2018 were included in the analysis. Exclusion criteria included childhood (<18 years), pancreatic transplantation procedure, and missing information on age or sex.

### Analysis Variables

The pancreatic disease type, pancreatic diagnosis role (main and/or side diagnosis), performed pancreatic resections, concomitant surgical procedures, and comorbidities were extracted for each treatment case. The pancreatic diagnosis type was classified as malignant neoplasm, benign neoplasm, acute pancreatitis, chronic pancreatitis, neoplasm of unclear dignity, or no pancreatic diagnosis. In our analysis, we consider the entire range of pancreatic resections (malignant, benign, chronic, and acute) because we are convinced that the experience of a center results from the entire range of indications for pancreatic resections. As this can also represent a source of bias, we also considered the resected pancreatic carcinomas as a subgroup to check whether the results also apply to this homogeneous group. For a more precise risk assessment, concomitant surgical resections, including gastric, small intestine, and visceral veins and arteries, were identified using appropriate medical procedure codes. Comorbidities that have been proven to affect inhospital mortality during pancreatic resections have been determined according to Quan-Elixhauser coding algorithms with minor adjustments to capture differences in the German modification of the International Classification of Diseases, 10th Revision system.^11,20^ Furthermore, dementia, cerebrovascular disease, myocardial infarction, and the differentiation between mild and moderate/severe liver disease, as specified by Charlson, were utilized, as they were not encompassed by the Elixhauser scheme.^20^ All codes for diagnosis and procedures are detailed in Supplemental Table S1, see http://links.lww.com/AOSO/A444.

The annual number of pancreatic resection procedures was calculated at the hospital level, accounting for multiple procedures on a single patient where appropriate. Next, HVPCs were identified, which performed at least 50 procedures per year throughout the observation period, which equates to approximately one procedure per week. We defined 50 pancreatic resections per year as the criterion for HVPCs because beyond this number of cases, there is no further statistically relevant reduction in hospital mortality (not shown).

### Statistical Analyses

The focus of the study was to evaluate the difference between crude and adjusted estimates of inhospital mortality. As discrepancies between crude and adjusted estimates within hospitals originated from different patient profiles, patient characteristics were compared between HVPC and non-HVPC groups.

Descriptive data analysis was performed by calculating mean, standard deviation, median, and interquartile range for continuous variables and absolute and relative frequencies for categorical variables. The analysis was conducted for the entire patient cohort and stratified by hospital group (HVPC vs non-HVPC). Additionally, we assessed the temporal trend in the proportion of procedures performed in HVPCs using the Cochran-Armitage trend test.

Crude inhospital mortality rates were computed for each hospital within the HVPC group. To determine risk-adjusted mortality rates, a generalized mixed model was applied using the lme4 package,^21^ with hospital as random effect and inhospital mortality as outcome (logit link). Fixed effects included age group, sex, pancreatic diagnosis group, resection procedure, admission condition, presence of specific comorbidities, performed concomitant resections, and year of surgery. Parameter estimation was conducted using the Laplace approximation, resolving nonconvergence issues by excluding variables with the highest variance inflation factors until convergence was achieved. Model performance was assessed using conditional $R^{2}$ and c-statistic. The intraclass correlation coefficient was determined as a measure of how much variation in the outcome can be attributed to the hospital level. The suitability of the model with random intercept was compared with the model without, using a likelihood ratio test.

To obtain the hospital-specific risk-adjusted mortality rates, the procedure described by Pouw et al^22^ was used. Essentially, the number of observed deaths in a single hospital was divided by the sum of the predicted probabilities of that hospital and then multiplied by the overall mortality rate of the entire population. Differences in crude and adjusted mortality rates have been depicted graphically and analyzed statistically by the computation of 95% confidence intervals (CIs) for the difference. The impact of a distinct factor on inpatient mortality was assessed through estimated marginal means.

To investigate how case mix disparities between HVPCs and non-HVPCs influence hospital mortality, we utilized the following method. First, we calculated the difference in patient proportions for each mortality factor between the groups. We multiplied the relative differences by the total number of patients in the HVPC group to determine the number of cases that would have been treated additionally or less if the HVPCs had treated the same case mix as the non-HVPCs. Next, we determined the impact on mortality by calculating the difference between the estimated mortality of the respective category and the reference category. Finally, we determined the impact on case fatality by calculating the product of the discrepancy in case numbers and the variance in mortality rates.

A subgroup analysis was conducted to investigate the robustness of the results for a homogeneous patient population. This involved repeating the previously described analyses exclusively for elective patients with malignant pancreatic carcinoma.

Remote data processing and all statistical analyses were performed using R 4.2.3 (The R Foundation, Vienna, Austria). All tests were two-tailed and statistical significance was set at a *P*-value threshold of less than 0.05. As only secondary data were analyzed, ethical approval was not required by German law. The study was registered at ClinicalTrials.gov under NCT06390891. Reporting of the study follows the strengthening the reporting of observational studies in epidemiology guideline.^23^

## RESULTS

During the analysis period, a total of 86,073 patients underwent pancreatic resection across 921 hospitals. An additional 315 patients received surgical treatment during this period, but they were excluded from the analysis due to specific exclusion criteria, primarily related to age (n = 273 patients under 18 years) or combination procedures involving pancreas transplantations.

Only 23 hospitals performed at least 50 pancreatic resections per year and were categorized as HVPCs. These hospitals served 24.2% of all patients (n = 20,798), a proportion that remained constant throughout the observation period ($\chi^{2}\left(df=7\right)=5.79,P=0.56$). Table 1 presents the patient characteristics of the entire cohort and stratified by hospital grouping. The crude inhospital mortality rate for all patients undergoing resection was 9.0%. The crude hospital mortality rate was notably lower in the HVPC group (5.5%) compared with the non-HVPC group (10.1%) (univariate analysis: $\chi^{2}\left(df=1\right)=403.2,P<0.001$). The comparison of patient populations in both clinic groups showed that HVPCs treated younger and less morbid patients and had fewer emergency admissions. Only three comorbidity conditions (pulmonary circulation disorders, uncomplicated diabetes and coagulopathy) were more frequently observed in HVPCs (Table 1). In contrast, HVPCs performed more complex pancreatic procedures including vascular replacement surgery (Table 2).

**TABLE 1.** Patient Characteristics: Sex, Age, and Comorbidities

Variable/Value	Total	HVPC	Non-HVPC	Difference	Chi-Square Test
Number of patients	86,073 (100.0%)	20,798 (100.0%)	65,275 (100.0%)		
Inhospital deaths	7,766 (9.0%)	1,154 (5.5%)	6612 (10.1%)	−953 (−4.6%)	P < 0.001
Sex					P = 0.211
Male	47,126 (54.8%)	11,309 (54.4%)	35,817 (54.9%)	−103 (−0.5%)	
Female	38,947 (45.2%)	9,489 (45.6%)	29,458 (45.1%)	103 (0.5%)	
Age group					P < 0.001
18–49	9322 (10.8%)	2885 (13.9%)	6437 (9.9%)	834 (4.0%)	
50–59	16,172 (18.8%)	4239 (20.4%)	11,933 (18.3%)	437 (2.1%)	
60–69	22,504 (26.1%)	5705 (27.4%)	16,799 (25.7%)	352 (1.7%)	
70–79	30,312 (35.2%)	6683 (32.1%)	23,629 (36.2%)	−846 (−4.1%)	
≥80	7763 (9.0%)	1286 (6.2%)	6477 (9.9%)	−778 (−3.7%)	
Comorbidity					
Congestive heart failure	6898 (8.0%)	975 (4.7%)	5923 (9.1%)	−912 (−4.4%)	P < 0.001
Cardiac arrhythmias	3147 (3.7%)	727 (3.5%)	2420 (3.7%)	−44 (−0.2%)	P = 0.156
Pulmonary circulation disorders	2133 (2.5%)	658 (3.2%)	1475 (2.3%)	188 (0.9%)	P < 0.001
Peripheral vascular disorders	6434 (7.5%)	1422 (6.8%)	5012 (7.7%)	−175 (−0.8%)	P < 0.001
Hypertension, uncomplicated	43,362 (50.4%)	9569 (46.0%)	33,793 (51.8%)	−1.198 (−5.8%)	P < 0.001
Hypertension, complicated	2709 (3.1%)	284 (1.4%)	2425 (3.7%)	−489 (−2.3%)	P < 0.001
Paralysis	1123 (1.3%)	216 (1.0%)	907 (1.4%)	−73 (−0.4%)	P < 0.001
Other neurological disorders	2381 (2.8%)	503 (2.4%)	1878 (2.9%)	−95 (−0.5%)	P < 0.001
Chronic pulmonary disease	7909 (9.2%)	1744 (8.4%)	6165 (9.4%)	−220 (−1.1%)	P < 0.001
Diabetes, uncomplicated	28,923 (33.6%)	7737 (37.2%)	21,186 (32.5%)	987 (4.7%)	P < 0.001
Hypothyroidism	10,706 (12.4%)	2283 (11.0%)	8423 (12.9%)	−401 (−1.9%)	P < 0.001
Renal failure	7530 (8.7%)	1266 (6.1%)	6264 (9.6%)	−730 (−3.5%)	P < 0.001
Solid tumor without metastasis	18,522 (21.5%)	3930 (18.9%)	14,592 (22.4%)	−719 (−3.5%)	P < 0.001
Coagulopathy	23,318 (27.1%)	6130 (29.5%)	17,188 (26.3%)	654 (3.1%)	P < 0.001
Obesity	6161 (7.2%)	1390 (6.7%)	4771 (7.3%)	−130 (−0.6%)	P = 0.002
Deficiency anemias	1708 (2.0%)	360 (1.7%)	1348 (2.1%)	−70 (−0.3%)	P = 0.003
Alcohol abuse	3177 (3.7%)	649 (3.1%)	2528 (3.9%)	−156 (−0.8%)	P < 0.001
Dementia	716 (0.8%)	88 (0.4%)	628 (1.0%)	−112 (−0.5%)	P < 0.001
Cerebrovascular disease (C)	2235 (2.6%)	448 (2.2%)	1787 (2.7%)	−121 (−0.6%)	P < 0.001
Moderate or severe liver disease (C)	3390 (3.9%)	746 (3.6%)	2644 (4.1%)	−96 (−0.5%)	P = 0.003
Mild liver disease (C)	6336 (7.4%)	1346 (6.5%)	4993 (7.6%)	−248 (−1.2%)	P < 0.001

HVPCs perform at least 50 pancreatic resections annually. Comorbidities according to Elixhauser and Charlson (C) with evidence of impact on inhospital mortality.

**TABLE 2.** Treatment Characteristics: Admission Type, Diagnosis, Resection, and Concomitant Procedures

Variable/Value	Total	HVPC	Non-HVPC	Difference	Chi-Square Test
Number of patients	86,073 (100.0%)	20,798 (100.0%)	65,275 (100.0%)		
Admission type					*P* < 0.001
Regular	58,997 (68.5%)	16,076 (77.3%)	42,921 (65.8%)	2.400 (11.5%)	
Emergent	21,427 (24.9%)	2994 (14.4%)	18,433 (28.2%)	−2.879 (−13.8%)	
Relocated	5649 (6.6%)	1728 (8.3%)	3921 (6.0%)	479 (2.3%)	
Pancreatic diagnosis					*P* < 0.001
Malign neoplasm	47,957 (55.7%)	11,292 (54.3%)	36,665 (56.2%)	−390 (−1.9%)	
Benign neoplasm	6083 (7.1%)	1930 (9.3%)	4153 (6.4%)	607 (2.9%)	
Unclear dignity	2946 (3.4%)	928 (4.5%)	2018 (3.1%)	285 (1.4%)	
Acute pancreatitis	5231 (6.1%)	1028 (4.9%)	4203 (6.4%)	−311 (−1.5%)	
Chronic pancreatitis	8475 (9.8%)	2493 (12.0%)	5982 (9.2%)	587 (2.8%)	
Other pancreatic diagnosis	3504 (4.1%)	846 (4.1%)	2658 (4.1%)	−1 (0.0%)	
No pancreatic diagnosis	11,877 (13.8%)	2281 (11.0%)	9596 (14.7%)	−776 (−3.7%)	
Pancreatic diagnosis type					*P* < 0.001
Main diagnosis	35,763 (41.5%)	8368 (40.2%)	27,395 (42.0%)	−361 (−1.7%)	
Main and side diagnosis	28,889 (33.6%)	7983 (38.4%)	20,906 (32.0%)	1.322 (6.4%)	
Side diagnosis	9544 (11.1%)	2166 (10.4%)	7378 (11.3%)	−185 (−0.9%)	
No pancreatic diagnosis	11,877 (13.8%)	2281 (11.0%)	9596 (14.7%)	−776 (−3.7%)	
Resection procedure					*P* < 0.001
Distal pancreatectomy	21,946 (25.5%)	5431 (26.1%)	16,515 (25.3%)	169 (0.8%)	
Pancreaticoduodenectomy	49,535 (57.5%)	11,487 (55.2%)	38,048 (58.3%)	−636 (−3.1%)	
Duodenum-preserving pancreaticoduodenectomy	6295 (7.3%)	1449 (7.0%)	4846 (7.4%)	−95 (−0.5%)	
Total pancreatectomy	8298 (9.6%)	2431 (11.7%)	5867 (9.0%)	562 (2.7%)	
Concomitant procedures					
Gastric resection	3552 (4.1%)	673 (3.2%)	2879 (4.4%)	−244 (−1.2%)	*P* < 0.001
Small intestine resection	10,158 (11.8%)	2325 (11.2%)	7833 (12.0%)	−171 (−0.8%)	*P* = 0.001
Vena portae resections	3532 (4.1%)	1434 (6.9%)	2098 (3.2%)	766 (3.7%)	*P* < 0.001
Vena portae suture	2678 (3.1%)	1296 (6.2%)	1382 (2.1%)	856 (4.1%)	*P* < 0.001
Vena mesenterica superior suture	2264 (2.6%)	931 (4.5%)	1333 (2.0%)	506 (2.4%)	*P* < 0.001
Arteria mesenterica superior resection	115 (0.1%)	25 (0.1%)	90 (0.1%)	−4 (0.0%)	*P* = 0.543
Arteria hepatica resection	498 (0.6%)	174 (0.8%)	324 (0.5%)	71 (0.3%)	*P* < 0.001
Arteria hepatica suture	921 (1.1%)	293 (1.4%)	628 (1.0%)	93 (0.4%)	*P* < 0.001
Dialysis procedure	4,414 (5.1%)	908 (4.4%)	3506 (5.4%)	−209 (−1.0%)	*P* < 0.001
Endoscopic biliary drainage	3642 (4.2%)	1086 (5.2%)	2556 (3.9%)	272 (1.3%)	*P* < 0.001
Splenectomy	21,135 (24.6%)	5171 (24.9%)	15,964 (24.5%)	85 (0.4%)	*P* = 0.236

HVPCs with at least 50 resection procedures annually.

**FIGURE 1.**
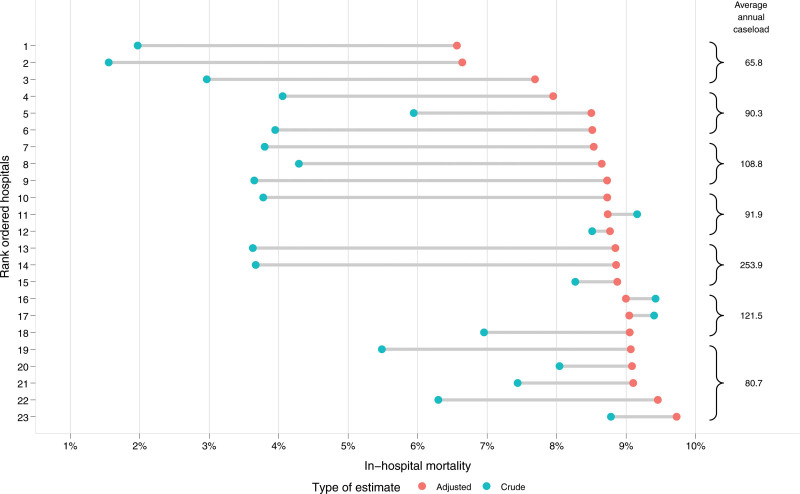
Inhospital mortality in HVPCs. Comparison of crude and adjusted inhospital mortality rates of 23 HVPCs. Average annual caseload refers to the group of centers marked with a curly bracket. Mortality was adjusted for age, sex, pancreatic diagnosis group, resection procedure, admission condition, comorbidities, concomitant resections, and year of surgery. HVPCs with at least 50 resections per year between 2011 and 2018.

The results of the logistic regression model, which was subsequently used to calculate risk-adjusted mortality rates, are shown in Supplementary Table 2, see http://links.lww.com/AOSO/A444. Table 2 also displays the estimated marginal means of inhospital mortality for the mortality factors. Model performance was good (c-statistic = 0.90, $R_{conditional}^{2}=0.53$) and the model with hospital random intercept was more appropriate than the model without ($\chi^{2}\left(df=1\right)=1009,P<0.001$), exhibiting an intraclass correlation coefficient of 0.091. Figure 1 displays the crude and risk-adjusted mortality rates for each HVPC. For privacy reasons, patient numbers are only displayed in groups of 3 to 5 HVPCs (braces). The average adjusted rate of inhospital mortality in the HVPC was 8.7%, which is 1.6 times higher than the unadjusted estimate. In 20 of 23 HVPCs, the risk-adjusted estimates exceeded the unadjusted mortality rates.

**FIGURE 2.**
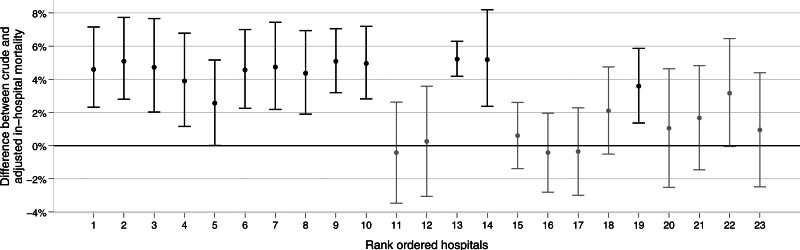
Difference between crude and adjusted mortality rates. The difference was calculated as the adjusted mortality rate minus the crude mortality rate. Values above zero indicate that the adjusted mortality rate was higher than the crude rate. Error bars represent the 95% confidence interval of the difference. A confidence interval including zero indicates a significant difference and is shown in black, otherwise in dark gray.

The difference between the two mortality rates, calculated as adjusted rate subtracted by crude rate, is depicted per HVPC in Figure 2. The error bars specify the 95% CI of the difference between the inhospital mortality rates. For 13 HVPCs, the difference between the two estimates is statistically significant as indicated by 95% CIs that did not include zero.

**FIGURE 3.**
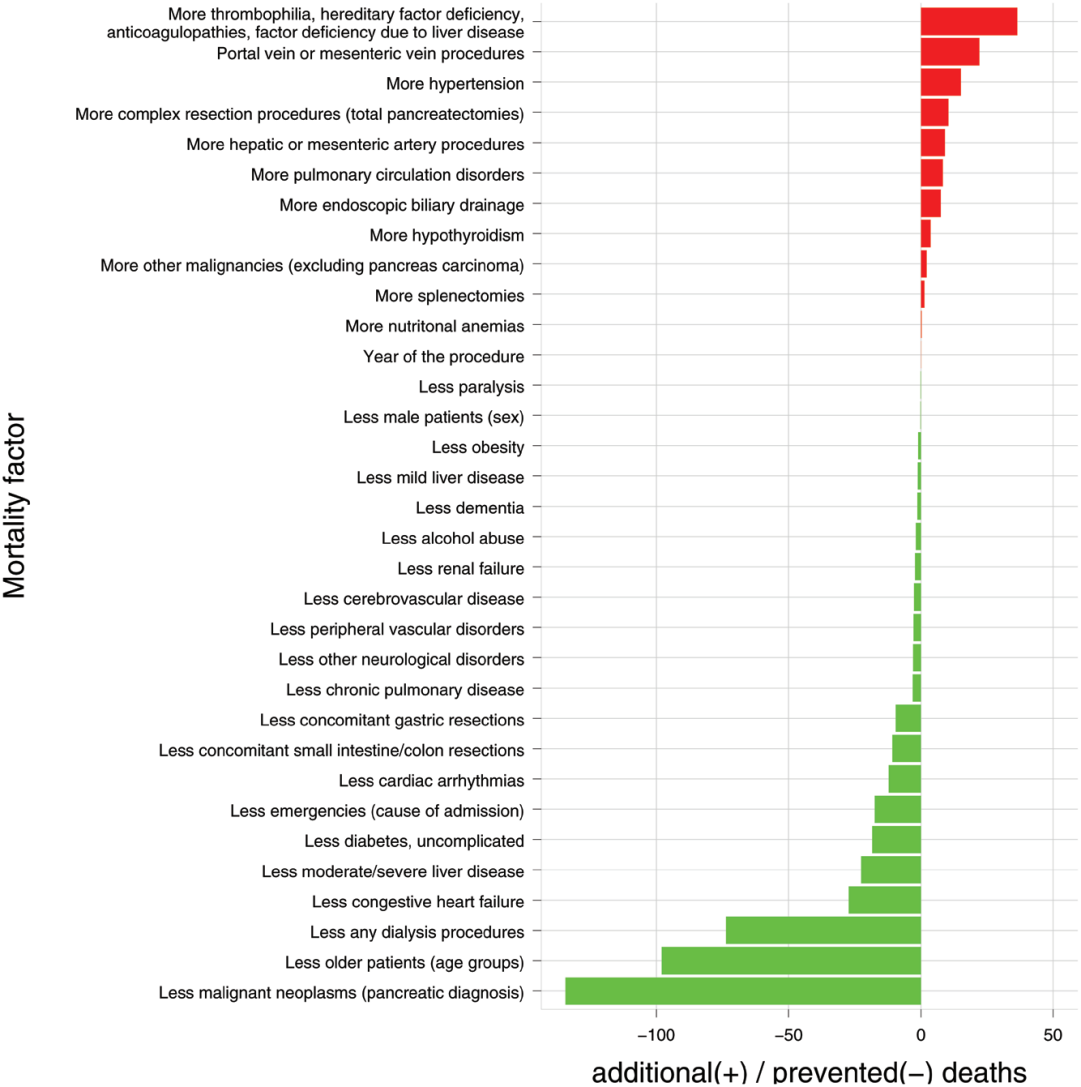
Combined effect of case-mix differences and mortality. Factors (patient characteristics, procedures) that are responsible for more deaths (red) or fewer deaths (green) in HVPCs compared with non-HVPCs. For example, in HVPCs more procedures on the portal vein or the mesenteric vein were performed, resulting in more deaths than in non-HVPCs. On the other hand, in HVPCs fewer patients with liver diseases or who were on dialysis were operated, which led to fewer deaths.

The impact of case-mix differences between HVPCs and non-HVPCs and the related differences in inhospital mortality are illustrated in Figure 3. In HVPCs, fewer patients aged ≥80 underwent resection, a factor linked to a high probability of inhospital mortality of 10.5% (vs 1.4% in the lowest age group). The reduced number of older patients of 778, combined with an increase in mortality of 9.1 percentage points, prevented about 71 deaths. Another example is the comorbidity of coagulopathy, which was more commonly treated in HVPCs and was associated with a 5-percentage point higher mortality rate than in patients without coagulopathy, resulting in additional deaths. Summarizing the effects across all mortality factors, 358 deaths were prevented due to the different case mix.

The subgroup analysis for elective patients with pancreatic carcinoma (n = 35,840) yielded comparable results regarding the impact of case-mix differences between HVPCs and non-HVPCs (Supplementary Fig. S1, see http://links.lww.com/AOSO/A444). However, due to the smaller number of patients in the subgroup, the estimated number of prevented deaths was lower, with a total of 159.

## DISCUSSION

The present observational, register-based, nationwide retrospective study analyzed patient characteristics and mortality rates of HVPCs with at least 50 pancreatic resections per year. The principal distinction between this study and numerous previous investigations is that the present study did not seek to contrast low-volume with high-volume hospitals. Rather, the objective was to compare the crude and adjusted mortality rates within the cohort of HVPCs. The objective of this analysis was to gain insights into the influence of potential discrepancies in patient characteristics between hospitals with disparate caseloads on the quality of outcomes. The discrepancy between crude and adjusted estimates can be attributed to variations in patient and treatment characteristics. The study is of great importance due to its analysis of over 86,000 pancreatic resections in a total population (Germany) over 8 years. This enables a comprehensive understanding of patient care and the quality of hospital outcomes. The analysis yielded four key findings: (a) HVPCs have a notably lower unadjusted inhospital mortality rate following pancreatic resection compared with non-HVPCs (5.5% and 10.1%, respectively); (b) procedures involving portal vein or arterial resections are performed twice as frequently in HVPCs than in non-HVPCs, with statistical significance; (c) the patients treated in HVPCs were significantly younger and had significantly fewer secondary diagnoses than in non-HVPCs; and (d) after adjustment, hospital mortality increased in 20 of 23 HVPCs as a result of incorporating patient characteristics in the analysis. In summary, the appropriate indication and selection of patients eligible for pancreatic resection represent an essential quality feature of large centers (HVPCs).

In accordance with prior research, a significant association was observed between caseload and hospital mortality.^1,3,5,9,10^ The mean hospital mortality rate was 5.5% in HVPCs and 10.1% in non-HVPCs. This finding lends support to the argument for further centralization of pancreatic surgery. It is questionable whether the minimum annual caseload of 20 procedures per hospital, which will come into effect in Germany in 2025, will result in a significant decrease in hospital mortality to the level of HVPCs. However, it is worth noting that a relevant number of clinics have low inhospital mortality but perform fewer than 50 pancreatic resections annually.^24^

Our analysis reveals that unadjusted hospital mortality rates in HVPCs exhibit considerable variation, ranging from 1.6% to 9.4%. Even after adjustment, the observed mortality rates remained highly variable, with mortality rates between 5.6% and 9.0%. This highlights the need to investigate the distinctive characteristics of HVPCs compared with non-HVPCs, and the potential causes for the observed variation within HVPCs. The present study aims to elucidate the differences between HVPCs and non-HVPCs.

Given the higher caseload and the associated increased expertise, it was to be expected that more complex cases would be treated in HVPCs than in non-HVPCs. The proportion of these complex cases was higher in HVPCs than in non-HVPCs, including total pancreatectomy (11.7% vs 9.0%) and vena portae resections (6.9% vs 3.2%). Despite the observation that the treatment of such complex cases results in higher hospital mortality (Fig. 3), mortality rates were significantly lower in the HVPCs than in the non-HVPCs, as previously mentioned. Furthermore, it has been demonstrated that patients in HVPCs were notably younger and had fewer mortality-relevant secondary diagnoses compared with those in non-HVPCs. This applies to 14 of the 21 analyzed secondary diagnoses that significantly impact hospital mortality rates in pancreatic surgeries.^11^

Different explanations can be posited for this finding. First, large pancreas centers usually serve a broader geographic area, necessitating long-distance travel for many patients. The provision of treatment in more distant facilities can present an additional challenge, particularly for older individuals with multiple chronic conditions. A phenomenon that has been described in the literature as travel burden.^15,16,18,19^ In contrast, longer travel times are less of a burden for younger people with fewer comorbidities who are in better overall health. Consequently, in HVPCs, a larger proportion of patients with better overall health conditions are treated. While this study is unable to elucidate mechanisms or reasons behind hospital selection, the findings are consistent with the findings of other studies. These have shown that for high-risk cancer surgery, lower socioeconomic status (eg, unemployment, low educational attainment) and specific patient attributes (such as advanced age, being a single parent, belonging to a minority ethnicity) decrease the probability of receiving treatment in a high-volume center.^18,25^ Furthermore, sex, income, and insurance status have also been demonstrated to impact the likelihood of undergoing pancreatectomy at a high-volume center.^17^ Unfortunately, the dataset contains no data on the socioeconomic status, educational background, medical history, or other personal history of patients, which allowed a more detailed analysis of underlying causes that may impact access to high-volume providers. In the German healthcare system, patients are free to select their physician and medical facility of choice, with insurance coverage extending to all cases. Consequently, financial considerations should exert minimal influence on access to medical facilities with high patient volumes.

Second, the indication for pancreatic resections and the selection of patients for surgery are of paramount importance for hospital mortality. Large pancreas centers possess pertinent experience owing to the high volume of cases, which has a beneficial impact on hospital mortality rates. The impact of patient selection on the resection rates among pancreatic cancer patients cannot be determined through the examination of diagnosis-related group routine data, as used in our study. An international comparative study reported the lowest resection rates in healthcare systems with centralized care (USA, the Netherlands, Denmark) compared with noncentralized healthcare systems.^26^ Further analyses based on cancer registry data are necessary to investigate these issues.

To compare hospital outcomes, it is well established to adjust results for case severity.^13,14,22,27,28^ The findings reveal that the majority of HVPCs (13 of 23) exhibited significantly lower observed mortality rates than the adjusted mortality rates. In seven HVPCs, the adjusted values were slightly higher than the raw mortality rates, although this was not statistically significant. The adjusted mortality rates were below the raw values in only three HVPCs, although not significantly. This indicates that the adjusted inpatient death rate in all 23 HVPCs is at least comparable to, if not better than, the crude inpatient death rate. These findings align with previous research on the influence of high case numbers on surgical outcome quality.^9,10^

The adjusted mortality rate takes into account the case complexity and the risk factors involved, thereby providing an estimate of the expected mortality rate for the given case mix. If the actual case mix is less complex than anticipated, the adjusted rate may exceed the observed one. Hospitals that treat less risky patients may show lower observed mortality rates, thereby creating a discrepancy due to case complexity. This can be attributed to case selection bias, as observed in retrospective case–control studies.^27^ In our study, HVPC and non-HVPC patient characteristics differ, challenging the representativeness of cases and controls. This has implications for the assessment of hospital quality, underscoring the need to exercise caution when comparing institutions with diverse case mixes. Hospitals that treat severely ill patients may demonstrate higher observed and adjusted mortality rates, highlighting the challenges in managing complex cases. Adjusted results should be employed for meaningful comparisons; however, residual confounding may persist due to routine data limitations and a lack of crucial patient information such as Union Internationale Contre le Cancer stages.^11,28^

In 2002, Birkmeyer et al^1^ published analyses on outcome quality that demonstrated an association between hospital caseload and outcome quality. These methods have since been established as standard, with their results used for health system planning. Through further advancements, our team and other researchers have demonstrated the significance of conducting differentiated analysis in addition to mean value evaluation to gain a more comprehensive understanding of the processes that result in high-quality outcomes.^9,10,24^

Several limitations of the study need to be considered, to gain a full understanding of its findings. The principal limitation of the study is the inability of retrospective observational studies to ascertain causality. It is possible that the observed phenomena may be influenced by unidentified confounding factors. Further prospective studies are required to validate the conclusions. As the analysis is based on an administrative dataset, all the inherent shortcomings of this data type are taken into account. Primarily, missing clinical variables (eg, Union Internationale Contre le Cancer stage, ASA score, and histopathological data) that are important for assessing disease severity are not included. With these data, a more comprehensive and finer risk adjustment would have been possible. In addition, it is not possible to follow patients over several hospital stays, so a death after readmission or transfer could not be attributed to the original treating hospital. It is also possible that differences in discharge management between hospitals influence the results, as earlier discharges or transfers are associated with lower inhospital mortality.

Despite the limitations, the present study provides a comprehensive insight into the national healthcare situation, especially due to the scope of the data, which is practically equivalent to a complete census. Furthermore, it can be assumed that diagnoses and procedures were fully recorded, given the billing relevance of the interventions studied. It can also be assumed that there was no miscoding of the endpoint of inhospital mortality.

## CONCLUSIONS

Treatment in major pancreas centers with at least 50 resections per year is associated with lower mortality rates than in other hospitals. Patients with pancreatic surgery that involving the portal vein or visceral arteries procedures have significantly better outcomes when resected at HVPCs than in non-HVPCs. However, next to the higher surgical expertise in high-volume centers, the improved outcome may be attributed to superior health status of patients. Further studies are required to investigate whether this is caused by patient-sided selection, as described in the context of the travel bias, or more appropriate indication and selection of patients eligible for pancreatic resection have to be investigated by further studies.

## Supplementary Material


