# Supplementary material for: Not Only Caseload but Also Patient Selection Is Predictive of Mortality After Pancreatic Resection
Source: Ann Surg Open. 2024 Dec 30;6(1):e536. doi: 10.1097/AS9.0000000000000536 (PMC11932614; doi:10.1097/AS9.0000000000000536)
Supplement: Supplementary file 1 [file as9-6-e536-s001.pdf]

Supplement

Table S1: Procedure definitions

Definition	Condition	Procedure/Diagnosis codes
Inclusion		
	Distal pancreatectomy	5-524.0
	Pancreaticoduodenectomy	5-524.1, 5-524.2
	Duodenum-preserving pancreaticoduodenectomy	5-524.3, 5-524.4, 5-524.x, 5-524.y
	Total pancreatectomy	5-525.0, 5-525.1, 5-525.2
Diagnoses		
	Acute pancreatitis	K85
	Chronic pancreatitis	K86.0, K86.1
	Malignant neoplasms	C24.1, C25
	Benign neoplasms	D13.6, D13.7
	Neoplasms of uncertain/unclear behavior	D37.70
	Other	K86.2, K86.3, K86.8, K86.8, K86.9
Exclusion procedures		
	Removal of a pancreas graft	5-525.3
	Postmortem pancreatectomy (for transplantation)	5-525.4
	Transplantation of pancreas (tissue)	5-528
Comorbidities		
	Congestive heart failure	I09.9, I11.0, I13.0, I13.2, I25.5, I42.0, I42.[5-9], I43, I50, P29.0
	Cardiac arrhythmias	I44.[1-3], I45.6, I45.9, I4[7-9], R00.[0|1|8], T82.1, Z45.0, Z95.0
	Pulmonary circulation disorders	I26, I27, I28.[0|8|9]
	Peripheral vascular disorders	I70, I71, I73.[1|8|9], I77.1, I79.[0|2], K55.[1|8|9], Z95.[8|9]
	Hypertension, uncomplicated	I10
	Hypertension, complicated	I11, I12, I13, I15
	Paralysis	G04.1, G11.4, G80.1, G80.2, G81, G82, G83.[0-4|9]
	Other neurological disorders	G1[0-3], G2[0-2], G25.[4|5], G31.[2|8|9], G32, G3[5-7], G4[0|1], G93.[1|4], R47.0, R56
	Chronic pulmonary disease	I27.[8|9], J4[0-7], J6[0-7], J68.4, J70.[1|3]
	Diabetes, uncomplicated	E1[0-4].[0|1|9]
	Hypothyroidism	E0[0-3], E89.0
	Renal failure	I12.0, I13.1, N18, N19, N25.0, Z49, Z94.0, Z99.2
	Solid tumor without metastasis	C0[0-9], C1[0-9], C2[0-3|6], C24[0|8|9], C3[0-4|7-9], C4[0|1|3|5-9], C5[0-8], C6[0-9], C7[0-6], C97
	Coagulopathy	D6[5-8], D69.[1|3-6]
	Obesity	E66
	Deficiency anemia	D50.[8-9], D5[1-3]
	Alcohol abuse	F10, E52, G62.1, I42.6, K29.2, K70.[0|3|9], T51, Z50.2, Z71.4, Z72.1
	Dementia	F0[0-3], F05.1, G30, G31.1
	Cerebrovascular disease (C)	G4[5|6], H34.0, I6[0-9]
	Moderate/severe liver disease (C)	I85, I86.4, I98.2, K70.4, K71.1, K72, K76.[5-7]
	Mild liver disease (C)	B18, K70[0-3|9], K71[3-5|7], K73, K74, K76[0|2-4|8|9], Z94.4

Definition	Condition	Procedure/Diagnosis codes
Concomitant procedures		
	Gastric resections	5-43[5-7]
	Small intestine resection	5-45[4-6]
	Vena portae resections	5-382.9d, 5-383.9d
	Arteria mesenterica superior resection	5-382.65, 5-383.65
	Vena portae suture	5-388.9d
	Vena mesenterica superior suture	5-382.9g, 5-383.9g
	Arteria hepatica resection	5-382.61, 5-383.61
	Arteria hepatica suture	5-388.61
	Dialysis procedure	8-85[3-5]
	Endoscopic biliary drainage	5-514.5, 5-513.5
	Splenectomy	5-413

Sub-classifications of respective codes are included. ICD-10-GM codes for comorbidities are reported using numeric ranges ("-") and alternatives ("|")

Table S2: Logistic regression model of in-hospital mortality

Coefficients of the logistic regression model and mortality rates. Odds ratio (OR); Confidence interval (CI); Estimated marginal mean (EMM).

Variable/Value	OR	OR 95%-CI	P	Mortality (EMM)	Mortality (EMM) 95%-CI
Sex					
male				4.2	3.9, 4.5
female	0.95	0.89, 1.02	0.2	4.0	3.7, 4.3
Age group					
18 - 49				1.4	1.2, 1.6
50 - 59	1.77	1.49, 2.11	<0.001	2.4	2.2, 2.7
60 - 69	2.87	2.43, 3.39	<0.001	3.9	3.5, 4.2
70 - 79	4.73	4.02, 5.56	<0.001	6.2	5.8, 6.7
≥ 80	8.36	7.01, 9.97	<0.001	10.5	9.6, 11.4
Admission type					
Regular				3.9	3.6, 4.2
Emergent	1.21	1.13, 1.30	<0.001	4.6	4.3, 5.0
Relocated	1.28	1.13, 1.44	0.026	4.9	4.3, 5.5
Pancreatic diagnosis type					
Main diagnosis				4.1	3.8, 4.4
Main and side diagnosis	0.86	0.80, 0.93	<0.001	3.5	3.2, 3.9
Side diagnosis	1.13	1.01, 1.26	0.029	4.6	4.1, 5.1
No pancreatic diagnosis	1.38	1.23, 1.55	<0.001	5.5	5.0, 6.2
Pancreatic diagnosis					
Malign neoplasm				8.7	8.5, 9.0
Benign neoplasm	0.43	0.31, 0.57	<0.001	3.8	3.2, 4.4
Unclear dignity	0.63	0.41, 0.83	<0.001	5.3	4.7, 5.9
Acute appendicites	2.39	2.23, 2.55	<0.001	19.8	19.2, 20.5
Chronic appendicites	0.51	0.36, 0.68	<0.001	4.3	3.9, 4.8
Other pancreatic diagnosis	1.03	0.88, 1.21	0.6	8.8	8.2, 9.5
No pancreatic diagnosis	1.32	1.16, 1.48	<0.001	13.1	12.7, 13.5
Resection procedure					
Pancreaticoduodenectomy (Whipple Procedure)				4.8	4.4, 5.1
Distal pancreatectomy	0.51	0.46, 0.56	<0.001	2.5	2.2, 2.7
Duodenum-preserving pancreaticoduodenectomy	0.75	0.66, 0.85	<0.001	3.6	3.2, 4.1
Total pancreatectomy	1.53	1.37, 1.71	<0.001	7.1	6.4, 7.9
Concomitant procedures					
Gastric resection	2.05	1.82, 2.31	<0.001	7.9	7.0, 8.9
Small intestine resection	2.94	2.72, 3.18	<0.001	10.0	9.2, 10.8
Vena portae resections	1.25	1.08, 1.44	0.003	5.0	4.3, 5.8
Arteria mesenterica superior resection	5.14	3.13, 8.43	<0.001	18.0	11.8, 26.5
Vena portae suture	1.23	1.03, 1.47	0.025	5.0	4.2, 5.9
Vena mesenterica superior suture	1.37	1.15, 1.63	<0.001	5.5	4.6, 6.5

Variable/Value	OR	OR 95%-CI	P	Mortality (EMM)	Mortality (EMM) 95%-CI
Arteria hepatica resection	2.54	1.90, 3.40	<0.001	9.8	7.4, 12.7
Arteria hepatica suture	2.65	2.13, 3.29	<0.001	10.1	8.2, 12.3
Dialysis procedure	17.1	15.6, 18.8	<0.001	38.8	36.3, 41.4
Biliary drainage	1.74	1.54, 1.97	<0.001	6.8	6.0, 7.7
Splenectomy	1.43	1.30, 1.56	<0.001	5.3	4.8, 5.8
Cholecystectomy	0.87	0.81, 0.93	<0.001	3.8	3.6, 4.1
Comorbidity					
Congestive heart failure	1.82	1.66, 1.99	<0.001	6.9	6.3, 7.6
Cardiac arrhythmias	1.68	1.56, 1.80	<0.001	6.2	5.7, 6.7
Pulmonary circulation disorders	2.20	1.91, 2.55	<0.001	8.5	7.3, 9.8
Peripheral vascular disorders	1.42	1.29, 1.57	<0.001	5.6	5.0, 6.2
Hypertension, uncomplicated	0.82	0.76, 0.87	<0.001	3.7	3.5, 4.0
Hypertension, complicated	0.72	0.62, 0.84	<0.001	3.0	2.6, 3.6
Paralysis	1.05	0.83, 1.32	0.7	4.3	3.4, 5.4
Other neurological disorders	1.82	1.58, 2.09	<0.001	7.1	6.2, 8.2
Chronic pulmonary disease	1.37	1.24, 1.51	<0.001	5.4	4.9, 6.0
Diabetes, uncomplicated	0.60	0.56, 0.65	<0.001	3.0	2.7, 3.2
Hypothyroidism	0.78	0.71, 0.86	<0.001	3.3	3.0, 3.7
Renal failure	1.08	0.98, 1.18	0.11	4.4	4.0, 4.9
Solid tumor without metastasis	0.93	0.84, 1.02	0.13	3.9	3.5, 4.3
Coagulopathy	2.98	2.78, 3.19	<0.001	8.7	8.1, 9.3
Obesity	1.21	1.07, 1.36	0.002	4.9	4.3, 5.5
Deficiency anemias	0.91	0.75, 1.11	0.4	3.8	3.1, 4.6
Alcohol abuse	1.32	1.12, 1.55	<0.001	5.3	4.5, 6.2
Dementia	1.31	1.03, 1.67	0.028	5.3	4.2, 6.7
Cerebrovascular Disease (C)	1.56	1.32, 1.84	<0.001	6.2	5.3, 7.3
Moderate or Severe Liver Disease (C)	9.49	8.52, 10.6	<0.001	27.1	24.8, 29.6
Mild Liver Disease (C)	1.13	1.01, 1.26	0.038	4.6	4.1, 5.1
Year					
2011				4.9	4.5, 5.4
2012	0.91	0.80, 1.02	0.11	4.5	4.0, 5.0
2013	0.88	0.78, 1.00	0.046	4.4	4.0, 4.8
2014	0.84	0.74, 0.94	0.004	4.1	3.7, 4.6
2015	0.82	0.73, 0.93	0.002	4.1	3.7, 4.5
2016	0.78	0.69, 0.88	<0.001	3.9	3.5, 4.3
2017	0.71	0.62, 0.80	<0.001	3.5	3.2, 3.9
2018	0.74	0.65, 0.84	<0.001	3.7	3.3, 4.1

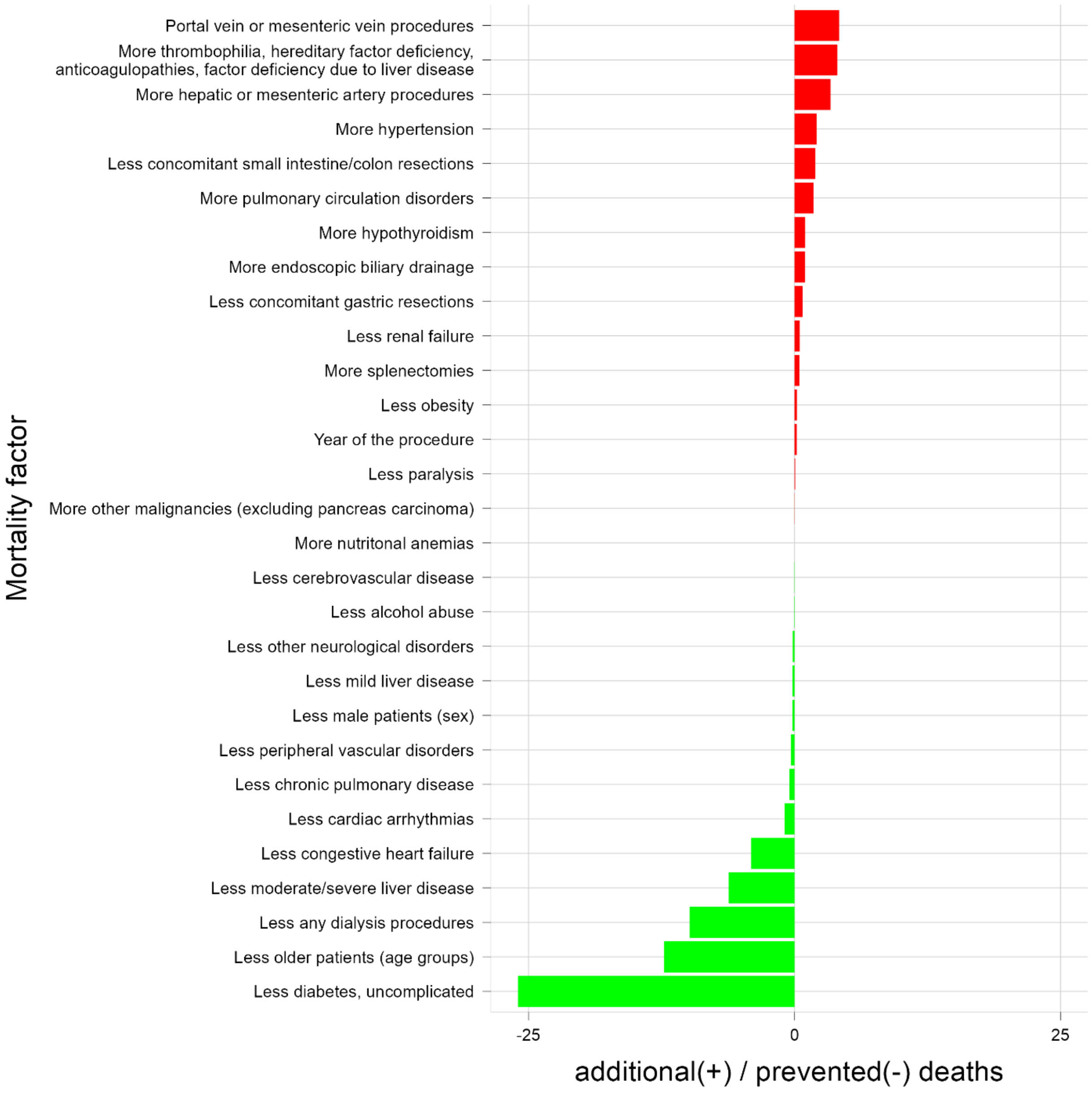

Figure S1: Combined effect of case-mix differences and mortality for elective patients with malign pancreatic carcinoma

Factors (patient characteristics, procedures) that are responsible for more deaths (red) or fewer deaths (green) in high volume pancreas centers (HVPCs) compared to non-HVPCs. For example, in HVPCs more procedures on the portal vein or the mesenteric vein were performed, resulting in more deaths than in non-HVPCs. On the other hand, in HVPCs fewer patients with liver diseases or who were on dialysis were operated, which led to fewer deaths.
